# (4b*S*,8a*S*)-1-Isopropyl-4b,8,8-trimethyl-4b,5,6,7,8,8a,9,10-octa­hydro­phenan­thren-2-yl acetate

**DOI:** 10.1107/S1600536814002748

**Published:** 2014-02-19

**Authors:** Radouane Oubabi, Aziz Auhmani, My Youssef Ait Itto, Abdelwahed Auhmani, Jean-Claude Daran

**Affiliations:** a Laboratoire de Synthése Organique et Physico-Chimie Moléculaire, Département de Chimie, Faculté des Sciences, Semlalia, BP 2390, Marrakech 40000, Morocco; b Laboratoire de Chimie de Coordination, 205 route de Narbonne, 31077 Toulouse Cedex 04, France

## Abstract

The hemisynthesis of the title compound, C_22_H_32_O_2_, was carried out through direct acetyl­ation reaction of the naturally occurring diterpene totarol [systematic name: (4b*S*,8a*S*)-4b,8,8-trimethyl-1-propan-2-yl-5,6,7,8a,9,10-hexa­hydro­phen­an­thren-2-ol]. The mol­ecule is built up from three fused six membered rings, one saturated and two unsaturated. The central unsaturated ring has a half-chair conformation, whereas the other unsaturated ring displays a chair conformation. The absolute configuration is deduced from the chemical pathway. The value of the Hooft parameter [−0.10 (6)] allowed this absolute configuration to be confirmed.

## Related literature   

For the synthesis, see: Short & Stromberg (1937 ▶). For biological properties of totarol, see: Barrero *et al.* (2003 ▶); Bernabeu *et al.* (2002 ▶); Haraguchi *et al.* (1996 ▶); Marcos *et al.* (2003 ▶); Tacon *et al.* (2012 ▶). For related structures, see: Zeroual *et al.* (2008 ▶); Pettit *et al.* (2004 ▶). For structural discussion, see: Cremer & Pople (1975 ▶); Flack (1983 ▶); Flack & Bernardinelli (2000 ▶); Spek (2009 ▶).
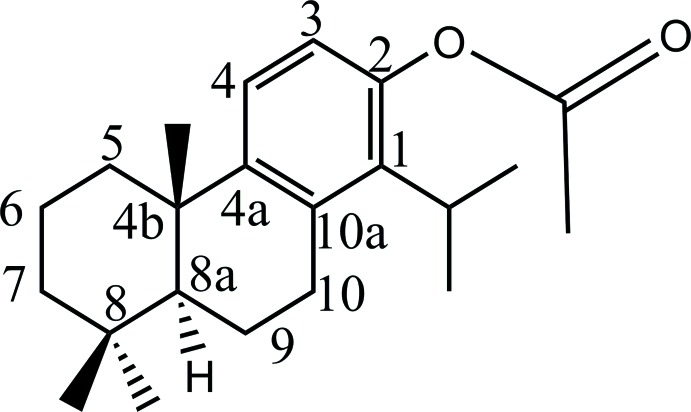


## Experimental   

### 

#### Crystal data  

C_22_H_32_O_2_
*M* *_r_* = 328.47
Monoclinic, 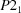

*a* = 7.4250 (2) Å
*b* = 10.5716 (3) Å
*c* = 12.0747 (3) Å
β = 90.124 (2)°
*V* = 947.79 (4) Å^3^
*Z* = 2
Cu *K*α radiation
μ = 0.55 mm^−1^
*T* = 180 K
0.38 × 0.38 × 0.14 mm

#### Data collection  

Agilent Xcalibur (Eos, Gemini ultra) diffractometer
Absorption correction: multi-scan (*CrysAlis PRO*; Agilent, 2012) ▶ *T*_min_ = 0.860, *T*_max_ = 1.000
4370 measured reflections
2511 independent reflections
2490 reflections with *I* > 2σ(*I*)
*R*_int_ = 0.012
θ_max_ = 60.7°

#### Refinement  

*R*[*F* ^2^ > 2σ(*F* ^2^)] = 0.029
*wR*(*F* ^2^) = 0.075
*S* = 1.04
2511 reflections
225 parameters
1 restraint
H-atom parameters constrained
Δρ_max_ = 0.13 e Å^−3^
Δρ_min_ = −0.14 e Å^−3^
Absolute structure: Refined as an inversion twin.
Absolute structure parameter: 0.0 (3)

### 

Data collection: *CrysAlis PRO* (Agilent, 2012 ▶); cell refinement: *CrysAlis PRO*; data reduction: *CrysAlis PRO*; program(s) used to solve structure: *SIR97* (Altomare *et al.*, 1999 ▶); program(s) used to refine structure: *SHELXL2013* (Sheldrick, 2008 ▶); molecular graphics: *ORTEPIII* (Burnett & Johnson, 1996 ▶) *ORTEP-3 for Windows* (Farrugia, 2012 ▶); software used to prepare material for publication: *SHELXL2013*.

## Supplementary Material

Crystal structure: contains datablock(s) I, New_Global_Publ_Block. DOI: 10.1107/S1600536814002748/xu5767sup1.cif

Structure factors: contains datablock(s) I. DOI: 10.1107/S1600536814002748/xu5767Isup2.hkl

Supporting information file. DOI: 10.1107/S1600536814002748/xu5767Isup3.cml

CCDC reference:

Additional supporting information:  crystallographic information; 3D view; checkCIF report

## Figures and Tables

**Fig. 1.**
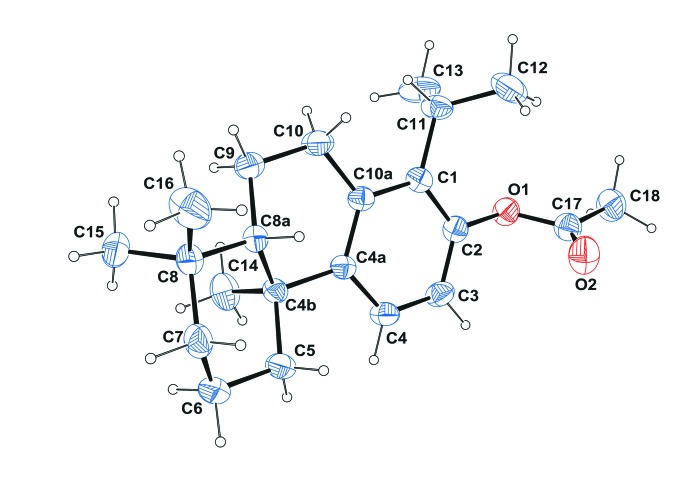
: Molecular view of compound (2) with the atom labeling scheme. Ellipsoids are drawn at the 50% probability level. H atoms are represented as small spheres of arbitrary radii.

## supplementary crystallographic information

### 1. Comment

The hemisynthesis of the title compound C_22_H_32_O_2_ 2 was carried out through direct acetylation reaction of naturally occurred Totarol (1). Totarol (1) is a naturally produced diterpene isolated from a several plants such as *Podocarpus totara* (Short & Stromberg, 1937) *Tetraclinis articulata* (Barrero *et al.*, 2003). It has been attracting great interest because of its biological properties ranging from Antimicrobial (Haraguchi *et al.*, 1996), anti-oxidant (Bernabeu *et al.*, 2002), Anti-inflammatory, analgesic, anti-tumoral (Marcos *et al.*, 2003) to Anti-plasmodial (Tacon *et al.*, 2012).

In the aim of preparing totarol derivatives, we report here, the hemisynthesis of (4b*S*,8a*S*)-1-isopropyl-4 b,8,8-trimethyl-4b,5,6,7,8,8a,9,10-octahydrophenanthren-2-yl acetate 2 from naturally occurred Totarol (1). Thus, treatment of (1) with acetic anhydride in pyridine provides (2) as colorless crystals in 97% yield. Its structure was fully characterized by its mass and NMR spectroscopic data. Furthermore, an X-ray single-crystal structure analysis allowed us to confirm unambiguously its full structure.

Compound (2) is built up from three fused six membered rings, a saturated one and two unsaturated (Fig. 1). The central unsaturated ring has an half chair conformation with puckering parameters: Q = 0.531 (2) Å, θ= 50.4 (2)° and φ= 120.1 (4)° (Cremer & Pople, 1975), whereas the second insaturated six-membered ring displays a chair conformation with puckering parameters: Q = 0.535 (3) Å, θ= 173.2 (3) (2)° and φ= 289 (2)°. Similar conformation for the three fused rings has been reported previously with hydroxyl substituent in place of the acetate in the title compound (Zeroual *et al.*, 2008) and with either an hydroxyl or a methoxy substituent on the central ring (Pettit *et al.*, 2004).

The absolute configuration (4*S*,8*S*) deduced from the chemical pathway is supported by the refinement of the Flack parameter, 0.0 (3), (Flack, 1983; Flack & Bernardinelli, 2000) and confirmed by the refinement of the Hooft parameter, -0.10 (6) (Spek, 2009).

### 2. Experimental

A solution of totarol (1) (90 mg, 0.314 mmol) in acetic anhydride (20 ml) and pyridine (20 ml) was heated under reflux for 24 h. After cooling, the mixture was acidified with 1N HCl solution then extracted with ether (3 × 20 ml). The organic layer was washed with water, dried on anhydrous Na_2_SO_4_ and then evaporated under reduced pressure. The obtained residue was chromatographied on silica gel column using hexane and ethyl acetate (97/3) as eluent, to give (4bS,8aS)-1-isopropyl-4 b,8,8-trimethyl-4 b,5,6,7,8,8a,9,10-octahydrophenanthren-2-yl acetate (2) (100 mg) in 97% yield.

### 3. Refinement

All H atoms attached to C atoms were fixed geometrically and treated as riding with C—H = 0.99 Å (methylene), 0.98 Å (methyl), 1.0 Å (methine) with *U*_iso_(H) = 1.2*U*_eq_(CH and CH_2_) or *U*_iso_(H) = 1.5*U*_eq_(CH_3_).

Although the standard deviation on the Flack's parameter, 0.0 (3), is rather high, the value of the Hooft's parameter, -0.10 (6), is more reliable and allows to confirm the absolute configuration. It is interesting to point out that inverting the configuration gives values of Hooft and Flack parameter close to 1.0 with similar standard deviation.

### Crystal data

**Table:**

C_22_H_32_O_2_	*F*(000) = 360
*M**_r_* = 328.47	*D*_x_ = 1.151 Mg m^−^^3^
Monoclinic, *P*2_1_	Cu *K*α radiation, λ = 1.54184 Å
*a* = 7.4250 (2) Å	Cell parameters from 3311 reflections
*b* = 10.5716 (3) Å	θ = 3.7–60.6°
*c* = 12.0747 (3) Å	µ = 0.55 mm^−^^1^
β = 90.124 (2)°	*T* = 180 K
*V* = 947.79 (4) Å^3^	Box, colourless
*Z* = 2	0.38 × 0.38 × 0.14 mm

### Data collection

**Table:**

Agilent Xcalibur (Eos, Gemini ultra) diffractometer	2511 independent reflections
Radiation source: Enhance Ultra (Cu) X-ray Source	2490 reflections with *I* > 2σ(*I*)
Mirror monochromator	*R*_int_ = 0.012
Detector resolution: 16.1978 pixels mm^-1^	θ_max_ = 60.7°, θ_min_ = 3.7°
ω scans	*h* = −7→8
Absorption correction: multi-scan Empirical absorption correction using spherical harmonics, implemented in SCALE3 ABSPACK scaling algorithm (*CrysAlis PRO*, Agilent, 2012)	*k* = −11→11
*T*_min_ = 0.860, *T*_max_ = 1.000	*l* = −13→13
4370 measured reflections	

### Refinement

**Table:**

Refinement on *F*^2^	H-atom parameters constrained
Least-squares matrix: full	*w* = 1/[σ^2^(*F*_o_^2^) + (0.0433*P*)^2^ + 0.168*P*] where *P* = (*F*_o_^2^ + 2*F*_c_^2^)/3
*R*[*F*^2^ > 2σ(*F*^2^)] = 0.029	(Δ/σ)_max_ < 0.001
*wR*(*F*^2^) = 0.075	Δρ_max_ = 0.13 e Å^−^^3^
*S* = 1.04	Δρ_min_ = −0.14 e Å^−^^3^
2511 reflections	Extinction correction: *SHELXL2013* (Sheldrick, 2013), Fc^*^=kFc[1+0.001xFc^2^λ^3^/sin(2θ)]^-1/4^
225 parameters	Extinction coefficient: 0.050 (2)
1 restraint	Absolute structure: Refined as an inversion twin.
Hydrogen site location: inferred from neighbouring sites	Absolute structure parameter: 0.0 (3)

### Special details

**Table:**

Experimental. Absorption correction: Empirical absorption correction using spherical harmonics, implemented in SCALE3 ABSPACK scaling algorithm (CrysAlis PRO; Agilent Technologies, 2012)
Geometry. All e.s.d.'s (except the e.s.d. in the dihedral angle between two l.s. planes) are estimated using the full covariance matrix. The cell e.s.d.'s are taken into account individually in the estimation of e.s.d.'s in distances, angles and torsion angles; correlations between e.s.d.'s in cell parameters are only used when they are defined by crystal symmetry. An approximate (isotropic) treatment of cell e.s.d.'s is used for estimating e.s.d.'s involving l.s. planes.
Refinement. Refined as a 2-component inversion twin.

### Fractional atomic coordinates and isotropic or equivalent isotropic displacement parameters (Å^2^)

**Table:**

	*x*	*y*	*z*	*U*_iso_*/*U*_eq_	
C1	0.6487 (3)	0.6992 (2)	0.17276 (16)	0.0270 (5)	
C2	0.4780 (3)	0.7203 (2)	0.13012 (17)	0.0283 (5)	
C3	0.3461 (3)	0.7823 (2)	0.18816 (19)	0.0334 (5)	
H3	0.2304	0.7948	0.1562	0.040*	
C4	0.3833 (3)	0.8262 (2)	0.29347 (18)	0.0319 (5)	
H4	0.2916	0.8682	0.3341	0.038*	
C4A	0.5533 (3)	0.81000 (19)	0.34131 (17)	0.0244 (5)	
C4B	0.5942 (3)	0.8675 (2)	0.45589 (18)	0.0258 (5)	
C5	0.4239 (3)	0.8711 (3)	0.52884 (19)	0.0346 (6)	
H5A	0.3376	0.9327	0.4972	0.041*	
H5B	0.3659	0.7868	0.5276	0.041*	
C6	0.4646 (3)	0.9074 (3)	0.6486 (2)	0.0406 (6)	
H6A	0.5144	0.9942	0.6509	0.049*	
H6B	0.3515	0.9067	0.6919	0.049*	
C7	0.5980 (3)	0.8167 (3)	0.70024 (18)	0.0371 (6)	
H7A	0.5427	0.7315	0.7033	0.045*	
H7B	0.6222	0.8438	0.7773	0.045*	
C8	0.7773 (3)	0.8075 (2)	0.63830 (18)	0.0338 (5)	
C8A	0.7386 (3)	0.7834 (2)	0.51303 (17)	0.0275 (5)	
H8A	0.6896	0.6954	0.5093	0.033*	
C9	0.9073 (3)	0.7813 (3)	0.44192 (19)	0.0372 (6)	
H9A	0.9521	0.8687	0.4322	0.045*	
H9B	1.0022	0.7319	0.4801	0.045*	
C10	0.8707 (3)	0.7233 (3)	0.32926 (18)	0.0366 (6)	
H10A	0.9617	0.7561	0.2768	0.044*	
H10B	0.8890	0.6307	0.3351	0.044*	
C10A	0.6862 (3)	0.74630 (19)	0.28005 (17)	0.0261 (5)	
C11	0.7945 (3)	0.6336 (2)	0.10479 (18)	0.0343 (6)	
H11	0.8978	0.6181	0.1562	0.041*	
C12	0.7413 (4)	0.5048 (3)	0.0572 (2)	0.0540 (8)	
H12A	0.8501	0.4562	0.0400	0.081*	
H12B	0.6704	0.5170	−0.0105	0.081*	
H12C	0.6691	0.4585	0.1117	0.081*	
C13	0.8634 (4)	0.7225 (3)	0.0150 (2)	0.0518 (7)	
H13A	0.9103	0.7998	0.0492	0.078*	
H13B	0.7644	0.7440	−0.0355	0.078*	
H13C	0.9597	0.6807	−0.0266	0.078*	
C14	0.6541 (4)	1.0050 (2)	0.4337 (2)	0.0433 (6)	
H14A	0.5590	1.0497	0.3933	0.065*	
H14B	0.7647	1.0047	0.3895	0.065*	
H14C	0.6768	1.0478	0.5044	0.065*	
C15	0.8902 (4)	0.9257 (3)	0.6616 (2)	0.0489 (7)	
H15A	0.9251	0.9270	0.7399	0.073*	
H15B	0.8193	1.0013	0.6444	0.073*	
H15C	0.9986	0.9243	0.6154	0.073*	
C16	0.8803 (4)	0.6933 (3)	0.6843 (2)	0.0561 (8)	
H16A	0.8152	0.6154	0.6657	0.084*	
H16B	0.8905	0.7008	0.7649	0.084*	
H16C	1.0009	0.6905	0.6516	0.084*	
C17	0.3222 (3)	0.5899 (2)	0.00169 (19)	0.0345 (6)	
C18	0.3010 (4)	0.5659 (3)	−0.1197 (2)	0.0460 (7)	
H18A	0.1878	0.5206	−0.1331	0.069*	
H18B	0.4021	0.5146	−0.1461	0.069*	
H18C	0.2991	0.6467	−0.1594	0.069*	
O1	0.4419 (2)	0.68523 (16)	0.01943 (11)	0.0338 (4)	
O2	0.2470 (2)	0.53449 (19)	0.07423 (14)	0.0491 (5)	

### Atomic displacement parameters (Å^2^)

**Table:**

	*U*^11^	*U*^22^	*U*^33^	*U*^12^	*U*^13^	*U*^23^
C1	0.0302 (12)	0.0261 (11)	0.0248 (11)	0.0012 (10)	0.0028 (9)	0.0023 (9)
C2	0.0305 (12)	0.0316 (12)	0.0230 (10)	−0.0026 (10)	0.0004 (9)	0.0014 (9)
C3	0.0244 (12)	0.0444 (13)	0.0315 (12)	0.0021 (11)	−0.0022 (9)	0.0002 (10)
C4	0.0269 (12)	0.0355 (12)	0.0333 (12)	0.0060 (10)	0.0031 (9)	−0.0010 (10)
C4A	0.0256 (11)	0.0205 (10)	0.0271 (11)	0.0010 (9)	0.0026 (8)	0.0024 (9)
C4B	0.0262 (11)	0.0221 (10)	0.0290 (11)	0.0006 (9)	0.0022 (9)	−0.0022 (9)
C5	0.0302 (12)	0.0422 (13)	0.0314 (12)	0.0063 (11)	0.0022 (10)	−0.0072 (10)
C6	0.0327 (13)	0.0533 (17)	0.0357 (13)	0.0027 (12)	0.0065 (10)	−0.0129 (12)
C7	0.0413 (14)	0.0448 (14)	0.0253 (11)	−0.0082 (12)	0.0023 (10)	−0.0068 (11)
C8	0.0342 (12)	0.0386 (13)	0.0286 (11)	0.0002 (11)	−0.0037 (9)	−0.0071 (10)
C8A	0.0269 (11)	0.0271 (11)	0.0286 (11)	−0.0019 (10)	−0.0001 (9)	−0.0041 (9)
C9	0.0276 (12)	0.0497 (14)	0.0343 (13)	0.0040 (11)	−0.0011 (9)	−0.0077 (11)
C10	0.0280 (12)	0.0513 (15)	0.0305 (12)	0.0089 (11)	0.0005 (10)	−0.0047 (11)
C10A	0.0261 (11)	0.0259 (11)	0.0263 (11)	0.0000 (9)	0.0031 (9)	0.0028 (9)
C11	0.0322 (12)	0.0457 (14)	0.0251 (11)	0.0109 (11)	0.0018 (9)	−0.0012 (10)
C12	0.0587 (19)	0.0487 (17)	0.0546 (17)	0.0163 (14)	0.0060 (14)	−0.0166 (13)
C13	0.0399 (15)	0.0696 (19)	0.0460 (15)	0.0121 (14)	0.0175 (12)	0.0119 (14)
C14	0.0599 (17)	0.0281 (13)	0.0421 (14)	−0.0045 (12)	−0.0035 (12)	0.0017 (11)
C15	0.0363 (14)	0.0662 (18)	0.0441 (15)	−0.0116 (13)	−0.0023 (11)	−0.0189 (14)
C16	0.068 (2)	0.0664 (18)	0.0338 (13)	0.0231 (17)	−0.0133 (13)	−0.0045 (14)
C17	0.0352 (13)	0.0364 (13)	0.0319 (12)	0.0016 (11)	−0.0017 (10)	−0.0013 (10)
C18	0.0489 (16)	0.0560 (17)	0.0332 (13)	−0.0039 (13)	−0.0023 (11)	−0.0089 (12)
O1	0.0341 (9)	0.0432 (9)	0.0239 (8)	−0.0036 (8)	−0.0002 (6)	−0.0011 (7)
O2	0.0587 (12)	0.0514 (11)	0.0372 (9)	−0.0160 (10)	0.0039 (9)	−0.0001 (9)

### Geometric parameters (Å, º)

**Table:**

C1—C2	1.385 (3)	C9—H9A	0.9900
C1—C10A	1.415 (3)	C9—H9B	0.9900
C1—C11	1.526 (3)	C10—C10A	1.512 (3)
C2—C3	1.372 (3)	C10—H10A	0.9900
C2—O1	1.412 (3)	C10—H10B	0.9900
C3—C4	1.381 (3)	C11—C13	1.524 (4)
C3—H3	0.9500	C11—C12	1.530 (4)
C4—C4A	1.398 (3)	C11—H11	1.0000
C4—H4	0.9500	C12—H12A	0.9800
C4A—C10A	1.406 (3)	C12—H12B	0.9800
C4A—C4B	1.541 (3)	C12—H12C	0.9800
C4B—C5	1.543 (3)	C13—H13A	0.9800
C4B—C14	1.544 (3)	C13—H13B	0.9800
C4B—C8A	1.553 (3)	C13—H13C	0.9800
C5—C6	1.526 (3)	C14—H14A	0.9800
C5—H5A	0.9900	C14—H14B	0.9800
C5—H5B	0.9900	C14—H14C	0.9800
C6—C7	1.512 (4)	C15—H15A	0.9800
C6—H6A	0.9900	C15—H15B	0.9800
C6—H6B	0.9900	C15—H15C	0.9800
C7—C8	1.532 (3)	C16—H16A	0.9800
C7—H7A	0.9900	C16—H16B	0.9800
C7—H7B	0.9900	C16—H16C	0.9800
C8—C15	1.530 (4)	C17—O2	1.193 (3)
C8—C16	1.533 (4)	C17—O1	1.361 (3)
C8—C8A	1.560 (3)	C17—C18	1.496 (3)
C8A—C9	1.520 (3)	C18—H18A	0.9800
C8A—H8A	1.0000	C18—H18B	0.9800
C9—C10	1.516 (3)	C18—H18C	0.9800
			
C2—C1—C10A	117.49 (18)	H9A—C9—H9B	108.0
C2—C1—C11	121.51 (18)	C10A—C10—C9	116.61 (19)
C10A—C1—C11	120.92 (19)	C10A—C10—H10A	108.1
C3—C2—C1	122.74 (18)	C9—C10—H10A	108.1
C3—C2—O1	118.32 (18)	C10A—C10—H10B	108.1
C1—C2—O1	118.77 (18)	C9—C10—H10B	108.1
C2—C3—C4	119.29 (19)	H10A—C10—H10B	107.3
C2—C3—H3	120.4	C4A—C10A—C1	120.89 (19)
C4—C3—H3	120.4	C4A—C10A—C10	120.43 (18)
C3—C4—C4A	121.18 (19)	C1—C10A—C10	118.65 (18)
C3—C4—H4	119.4	C13—C11—C1	110.0 (2)
C4A—C4—H4	119.4	C13—C11—C12	111.6 (2)
C4—C4A—C10A	118.38 (18)	C1—C11—C12	115.1 (2)
C4—C4A—C4B	119.92 (18)	C13—C11—H11	106.5
C10A—C4A—C4B	121.63 (18)	C1—C11—H11	106.5
C4A—C4B—C5	111.23 (17)	C12—C11—H11	106.5
C4A—C4B—C14	105.79 (18)	C11—C12—H12A	109.5
C5—C4B—C14	108.22 (19)	C11—C12—H12B	109.5
C4A—C4B—C8A	107.92 (17)	H12A—C12—H12B	109.5
C5—C4B—C8A	109.04 (17)	C11—C12—H12C	109.5
C14—C4B—C8A	114.6 (2)	H12A—C12—H12C	109.5
C6—C5—C4B	112.74 (18)	H12B—C12—H12C	109.5
C6—C5—H5A	109.0	C11—C13—H13A	109.5
C4B—C5—H5A	109.0	C11—C13—H13B	109.5
C6—C5—H5B	109.0	H13A—C13—H13B	109.5
C4B—C5—H5B	109.0	C11—C13—H13C	109.5
H5A—C5—H5B	107.8	H13A—C13—H13C	109.5
C7—C6—C5	111.0 (2)	H13B—C13—H13C	109.5
C7—C6—H6A	109.4	C4B—C14—H14A	109.5
C5—C6—H6A	109.4	C4B—C14—H14B	109.5
C7—C6—H6B	109.4	H14A—C14—H14B	109.5
C5—C6—H6B	109.4	C4B—C14—H14C	109.5
H6A—C6—H6B	108.0	H14A—C14—H14C	109.5
C6—C7—C8	114.08 (19)	H14B—C14—H14C	109.5
C6—C7—H7A	108.7	C8—C15—H15A	109.5
C8—C7—H7A	108.7	C8—C15—H15B	109.5
C6—C7—H7B	108.7	H15A—C15—H15B	109.5
C8—C7—H7B	108.7	C8—C15—H15C	109.5
H7A—C7—H7B	107.6	H15A—C15—H15C	109.5
C15—C8—C7	109.6 (2)	H15B—C15—H15C	109.5
C15—C8—C16	107.7 (2)	C8—C16—H16A	109.5
C7—C8—C16	107.9 (2)	C8—C16—H16B	109.5
C15—C8—C8A	114.3 (2)	H16A—C16—H16B	109.5
C7—C8—C8A	109.00 (17)	C8—C16—H16C	109.5
C16—C8—C8A	108.25 (19)	H16A—C16—H16C	109.5
C9—C8A—C4B	109.05 (18)	H16B—C16—H16C	109.5
C9—C8A—C8	113.59 (17)	O2—C17—O1	123.7 (2)
C4B—C8A—C8	117.57 (17)	O2—C17—C18	126.0 (2)
C9—C8A—H8A	105.2	O1—C17—C18	110.3 (2)
C4B—C8A—H8A	105.2	C17—C18—H18A	109.5
C8—C8A—H8A	105.2	C17—C18—H18B	109.5
C10—C9—C8A	111.51 (18)	H18A—C18—H18B	109.5
C10—C9—H9A	109.3	C17—C18—H18C	109.5
C8A—C9—H9A	109.3	H18A—C18—H18C	109.5
C10—C9—H9B	109.3	H18B—C18—H18C	109.5
C8A—C9—H9B	109.3	C17—O1—C2	117.79 (16)
